# Expectations, training and evaluation of intensive care staff to an interprofessional simulation course in Germany – Development of a relevant training concept

**DOI:** 10.3205/zma001302

**Published:** 2020-02-17

**Authors:** Markus Flentje, Lars Friedrich, Hendrik Eismann, Wolfgang Koppert, Heiner Ruschulte

**Affiliations:** 1 Hannover Medical School, Department of Anaesthesiology and Intensive Care Medicine, Hannover, Germany; 2 Sana Klinikum Hameln-Pyrmont, Anaesthesia and Intensive Care Medicine, Hameln, Germany

**Keywords:** interprofessional education, crisis resource management, intensive care unit, simulation

## Abstract

**Objective: **Increasingly, intensive care units (ICU) are operated by teams of physicians and nurses with specialist training in anaesthesia and intensive care. The aims of our study were to evaluate any prior experience, expectations and the requisites for interprofessional ICU simulation-based training (SBT), and to evaluate a newly designed training course incorporating these findings.

**Methods: **The study was laid out as a cross-sectional study and is projected in three steps. First, questionnaires were sent out to ICU nurses and physicians from 15 different hospitals in a greater metropolitan area (> million citizens). Based upon this survey a one-day ICU simulator course designed for 12 participants (6 nurses and 6 physicians) was developed, with evaluation data from four subsequent courses being analysed.

**Results: **In the survey 40% of nurses and 57% of the physicians had had prior exposure to SBT. Various course formats were explored with respect to duration, day of the week, and group composition. After completing the course, the majority deemed a full working day in interprofessional setting to be most appropriate (p<0.001). The scenarios were considered relevant and had a positive impact on communication, workflow and coping with stress.

**Conclusion: **Currently SBT is not a mainstream tool used by German ICU teams for further education, and this lack of familiarity must be taken into consideration when preparing SBT courses for them. We developed a nontechnical skills training course for ICU teams which was undertaken in the setting of simulated clinical scenarios (pertinent to their work environment). The participants found the course’s content to be relevant for their daily work, rated the course’s impact on their workplace practices as being good and advocated for longer training sessions.

## Zusammenfassung

**Hintergrund: **Auf Intensivstationen (ICU) arbeiten hoch spezialisierte Krankenpflegekräfte und Ärzte. Das Ziel dieser Arbeit war es daher, die Vorerfahrung dieses Personals zu interprofessionellen Simulationstrainings für Intensivstationen zu hinterfragen und Erwartungen an ein Trainingskonzept zu beschreiben. Der aus den Ergebnissen entstandene Trainingskurs wurde anschließend evaluiert.

**Methodik: **Die Studie war als Querschnittsstudie angelegt und wurde in drei Stufen durchgeführt. Zuerst wurden Fragebögen an Pflegepersonal und Ärzte von 15 Krankenhäusern unterschiedlicher Versorgungsstufen (>1 Millionen Einwohner Versorgungsgebiet) geschickt. Auf Basis der Ergebnisse wurde ein Simulationskurs mit dem Schwerpunkt „Arbeit auf der Intensivstation“ für je 12 Teilnehmer (6 Pflegekräfte und 6 Ärzte) entwickeln und schließlich mit Hilfe der Teilnehmer evaluiert.

**Ergebnisse:** In der Befragung gaben 40% des Pflegepersonals und 57% der Ärzte Vorerfahrungen mit Simulationstrainings an. Verschiedene Kursformate hinsichtlich Dauer, Veranstaltungstag und Zusammensetzung des Teilnehmerfeldes zur Bewertung vorgeschlagen. Die Teilnehmer bewerteten die Organisation als Ein-Tages-Kurs mit einer interprofessionellen Zusammensetzung als adäquat. Die vorgeschlagenen Szenarios wurden als relevant beurteilt und ein positiver Einfluss auf Kommunikation, Arbeitsabläufe und Stressverarbeitung angenommen.

**Schlussfolgerung: **Aktuell ist ein Simulationstraining kein regelhaft eingesetztes Trainingsinstrument auf deutschen Intensivstationen für die Weiter- und Fortbildung des Personals. Die geringe Erfahrung des Personals muss in die Trainingsplanung und Vorbereitung der Kurse berücksichtigt werden. Wir haben einen Trainingskurs mit Bezug zu non-technical Skills für Intensivstationen und klinisch relevanten Inhalten entwickelt. Die Teilnehmer werteten den Kurs als relevant für die tägliche Arbeit, schätzen den Einfluss auf Arbeitsabläufe positiv ein und wünschten sich längere Trainingseinheiten.

## Introduction

Professionals working in intensive care units (ICUs) manage patients with life threatening conditions from a multitude of different causes. This requires them to work in multi–professional (various job outlines) and multi–disciplinary (various specialist) teams that are dependent upon close interdisciplinary and interprofessional cooperation [1]. In healthcare interprofessional collaboration is not a “nice to have”, but is crucial for good medical care [2], [3]. Furthermore, ICU teams need to be regarded as high-responsibility teams [4] since their work environment is characterised by a high level of responsibility, an irreversibility of many therapeutic decisions and significant time pressure. The corollary of this work environment is that medical errors in patient care have been described to occur in 26.8% to 58% of ICU admissions [5], affecting both morbidity and mortality [6]. 

In addition the challenging working conditions, there is currently a shortage of nurses in Germany and more teamwork is one of many demands to improve the situation [7].

One of the many recommendations of the authors of “To Err is Human”, an early publication of identification of adverse events in patient care, was for the incorporation of interdisciplinary team training for their ICUs [8]. Medical errors are influenced by multiple factors including the patient safety culture [5], system design [9] and training programmes for the staff by means of both simulation and lecture [10]. Aviation, in comparison to healthcare, appreciated much earlier the influence effective nontechnical skills make, and as such ensured that training for their high–responsibility teams focused particularly upon the development of these skill sets as a part of safety culture (e.g. teamwork, task management, decision making and situational awareness). This has resulted in a 20-fold reduction in aviation accidents [11]. Similarly, improvements in anaesthesiologists’ nontechnical competencies and teamwork via simulation-based training has previously been demonstrated to have a positive influence on the quality of patient care in the operation theatre [12], [13]. Consequently, multiple recommendations have been given regarding the incorporation of these strategies into healthcare systems so as to help improve patient safety; including checklists [14], standardizing [8] and simulation-based human factors training [14]. Importantly, during 2010 the European Board of Anaesthesiology (EBA) and the European Society of Anaesthesiology (ESA) made a recommendation (as part of the Helsinki Declaration on patient safety) for the utilization of periodic simulation–based training [15]. Simulation is a generic term that can be defined as a context for learning that consists of a controlled and shielded representation of real-world situations, and a set of educational methods and procedures in which trainees feel simultaneously challenged and psychologically safe to practice and reflect on their performance [16]. In this study, we used the term simulation using high fidelity scenarios to improve non-technical skills.

The German Society of Anaesthesiology and Intensive Care Medicine (DGAI) successfully initiated a project to integrate simulation-based training into medical education in 2003 by offering every academic anaesthesiology department a patient simulator [8]. The vast majority accepted these simulators and started developing a variety of courses that addressed not only the needs of medical students, but also of other care providers [17]. To date, the actual number of programmes which were initiated and their respective participants (primarily from the non-academic institutions) are largely unknown. Simulation-based training (SBT) can be used didactically for the training of skills and knowledge, system analysis and team resource management [18]. For a highly effective training, an open-minded attitude towards training and the contents relevance for daily work is crucial [19]. Whilst organizing training courses for other German hospitals, we noticed that training goals were often not clearly defined. In the majority of these health care facilities, further education continues to be provided only by means of traditional lectures.

Curriculum development for medical education should be performed in a structured manner, to achieve a high efficiency. One of the well-known conceptual frameworks is the six-step approach described by Kern et al [20]. Table 1 (Tab. 1) shows the application of the six-step approach to the work conditions of intensive care unit in Germany. The open questions lead to the hypotheses of the study.

Our hypotheses were:

1. health care providers of an intensive care unit in Lower Saxony have little experience with simulation based training (SBT),
2. there are organizational framework conditions for the training that are preferred by the participants,
3. participants are not prepared for an interprofessional training and
4. there are relevant scenarios in the work environment of an intensive care unit to train non-technical skills.

## Materials and methods

### Research design

The study was planned as a cross-sectional study and is projected in three steps. First, to describe the previous experiences in SBT an initial survey was conducted. Then, based on the results, the training course were constructed. Most recently, an evaluation was conducted after health care providers participated in the course.

#### Initial survey: data collection

After formally gaining the permission from their relevant departmental heads, the medical and nursing staff from the ICU-teams in 15 different German hospital‘s (levels of treatment intensity ranging from standard care to maximum level intensive care) in the state of Lower Saxony were invited to participate in a survey regarding further education/ SBT in ICU. Because there is a mixed supply of hospital service in Germany (e.g. private, clerical, municipal), we were dependent on voluntary participation in the initial survey. The target hospitals had very different connections to our university hospital (e.g. former employees) and were chosen because of a presumed high willingness to participate (convenience sample).

We independently developed a questionnaire with 9 Items (passed through a local expert-round revision) to assess the current training practices, attitudes and expectations, as well as to identify potential topics of interest with regards to further education.

As demographic data, the profession of the participants (nurse, specialist ICU nurse, resident physician, board-certified physician), as well as the level of care of the participants hospital was registered. The item “previous experience with SBT” was assessed by means of a closed question (yes/no) as to whether the respondents had ever previously participated in a simulation course. The participants could rate their preferences either to train in interprofessional teams (physicians and nurses) or solely within their own occupational group. The envisioned effects of the training on non-technical skills like communication, workflow and coping with stressful situations were scored using a 6-point Likert scale (1=very strong effect; 6=no effect). “Participants’ needs and opinions” regarding potential scenario topics, that had been derived from established German anaesthesia training topics (a sepsis scenario was also added at the discretion of the authors, as it was deemed relevant for intensive care medicine), were evaluated using a 6-point Likert scale (1=very useful; 6=not at all useful). In addition, questions regarding the participant’s likely voluntariness, preferred duration of the training course and desired day of training were asked. These later questions were included to help us accommodate for a chronic organisational obstacle to staff participating in training courses in Germany, namely owing to further education being neither mandatory nor accounted for in standard working times. The printed questionnaires were distributed locally and were returned after completion either locally or by mail.

#### Development of simulation-based training course

Subsequently, an interprofessional SBT course for ICU-teams was developed using the results from the primary questionnaire in conjunction with the established SBT resources from the authors’ university-hospital (over 10 years of experience running, training and providing education in anaesthesia-related SBT courses). The trainings were “face-to-face” and took place in our hospital’s dedicated medical simulation centre. All of the instructors were required to minimally complete a four-day CRM-Instructor course (InFact® – local training concept in Germany). Based on our previous experience, we decided that each course would be attended by six ICU nurses and six physicians, all of whom were accustomed to working together in the same ICU. The first course was used as a pilot course to identify and then fix any unexpected difficulties as a result of this new course’s format, whilst ensuring that every instructor was comfortable with his or her tasks. No data material from this pilot course was used in the final analysis.

The material covered in each course was completed during an allotted eight-hour period (in accordance with the primary survey) and consisted of nine units/lessons. The first two units were: a theoretical introduction into crisis resource management (CRM), and an introduction to both the simulator (SimMan 3G, Laerdal Medical, Norway) and the working environment of the simulated ICU workplace (lessons 1-2). The application of the CRM principles, as per Gaba and Rall [22], were specified as learning targets (see table 2 (Tab. 2)). Thereafter, the participants performed six clinical scenarios (see table 3 (Tab. 3)) as a nurse-physician team under video surveillance (duration about 15 minutes), with each scenario being followed by a debriefing session (about 30 minutes) led by instructors from both professions (lessons 3-8). These scenarios were derived and developed independently by the authors from the highest rated themes/topics in the primary survey (one scenario was the result of combining two topics – “patient admission” and “shock” – after poor feedback regarding the “patient admission” scenario during the primary survey). Although the scenarios themselves were highly clinical and often involved significant but relevant technical aspects (so as to improve engagement and maximise their relevance for usual work practices), the primary focus of the debriefing sessions was related to the execution and role of NTS (non-technical skills) in each scenario. The final unit was a wrap-up session to summarize the experiences and then to return to “clinical reality”.

#### Data collection – Evaluation of the course

Four courses were subsequently given using this same format, and the accumulated information provided by 24 ICU nurses and 23 physicians was evaluated with respects to relevance, usefulness and the willingness to attend a similar course. After the course, all participants were asked again (by means of an 8 Item evaluation questionnaire) about the duration of the course, the kind of participation, the likely impact on their own department and the relevance of each scenario. In addition, an overall assessment of the course on a scale of 1 (very good) to 6 (fail) was requested. This style of scoring is the standard used in the German school system for grading, hence all of the participants were familiar with it. Likewise, the usefulness of the training for the participant’s daily work and their subjective feelings of embarrassment by watching colleagues and debriefing (6-Point-Likert scale, 1 (strongly agree) to 6 (strongly disagree) were assessed. As demographic data the profession of the participants was gathered (physician/ nurse).

Participation was voluntary and could be withdrawn at any time. The course was provided for free, without any expenses for participation. 

#### Data analysis

Excel-Software (Version 2010, Microsoft, Redmond, USA) was used for data collection and SPSS-Statistics 24 (IBM Corporation, USA) for the statistical analysis. All figures were created using GraphPad Prism 6.0h (GraphPad Software, Inc., USA). All data are presented in a descriptive manner. Our null hypothesis was that no difference between pre- and post-course survey would occur. Mann-Whitney-U tests for unpaired samples were conducted to compare the ratings pre and post training as pre- and post-training questionnaires were not matched. Chi-Square test were calculated to compare frequencies of the ratings for an estimated optimal course duration and the rating of the individual kind of participation. We assumed a p<0.05 as being statistically significant.

#### Ethical consideration

Due to the fact that the data collection and analysis was strictly anonymous there were no ethical doubts about the study. We consulted the local ethics committee at Hanover Medical School and received the decision, approval was not necessary.

## Results

Primary survey questionnaires were completed and returned by 207 respondents: 74 nurses, 44 specialist ICU nurses, 27 resident physicians, and 62 board-certified physicians. The treatment-level capabilities for the respondents’ institutions ranged from standard care (n=28), over advanced care (n=40) and maximum level care (e.g. university medical centre ICU, n=140). Forty percent of the nurses and 57% of the physicians had participated previously in simulation-based trainings.

In the initial survey, 27% of the nurses and 15% of the physicians indicated that they would prefer to undergo training in groups that were restricted to their own profession. As shown in table 4 (Tab. 4), the nurses indicated a preference for courses during normal weekdays, whereas the physicians had no clear preference for weekdays over weekends (Sundays were the least popular; and more than one day could be given as being desirable by each respondent).

Prior to participating in the course, 54% of the nurses considered the optimal duration of the course to be an entire working day (8 hours), 26% voted for 4 h and 15% for 2 h. While 46% of the physicians considered 8 h as optimal, 33% 4 h and 20% 2 h. After participating, 96% of the physicians considered a whole working day appropriate, as did 88% of the nurses (13% continued to prefer a 4 h duration). The increased preference for whole working day courses was significant (p<0.001).

In the initial survey, 1% of the nurses reported that they would not voluntarily participate in the training, whereas 69% would be happy to take part. Similarly, 4% of the physicians reported that they would not participate voluntarily, in comparison with 65% who would be happy to participate. 31% from each group were indifferent with regards to participating. After completing the course, 87% of the physicians and 79% of the nurses indicated that they would be happy to participate in a similar course again. The changes in willingness to participate were not significant.

**Figure 1:**
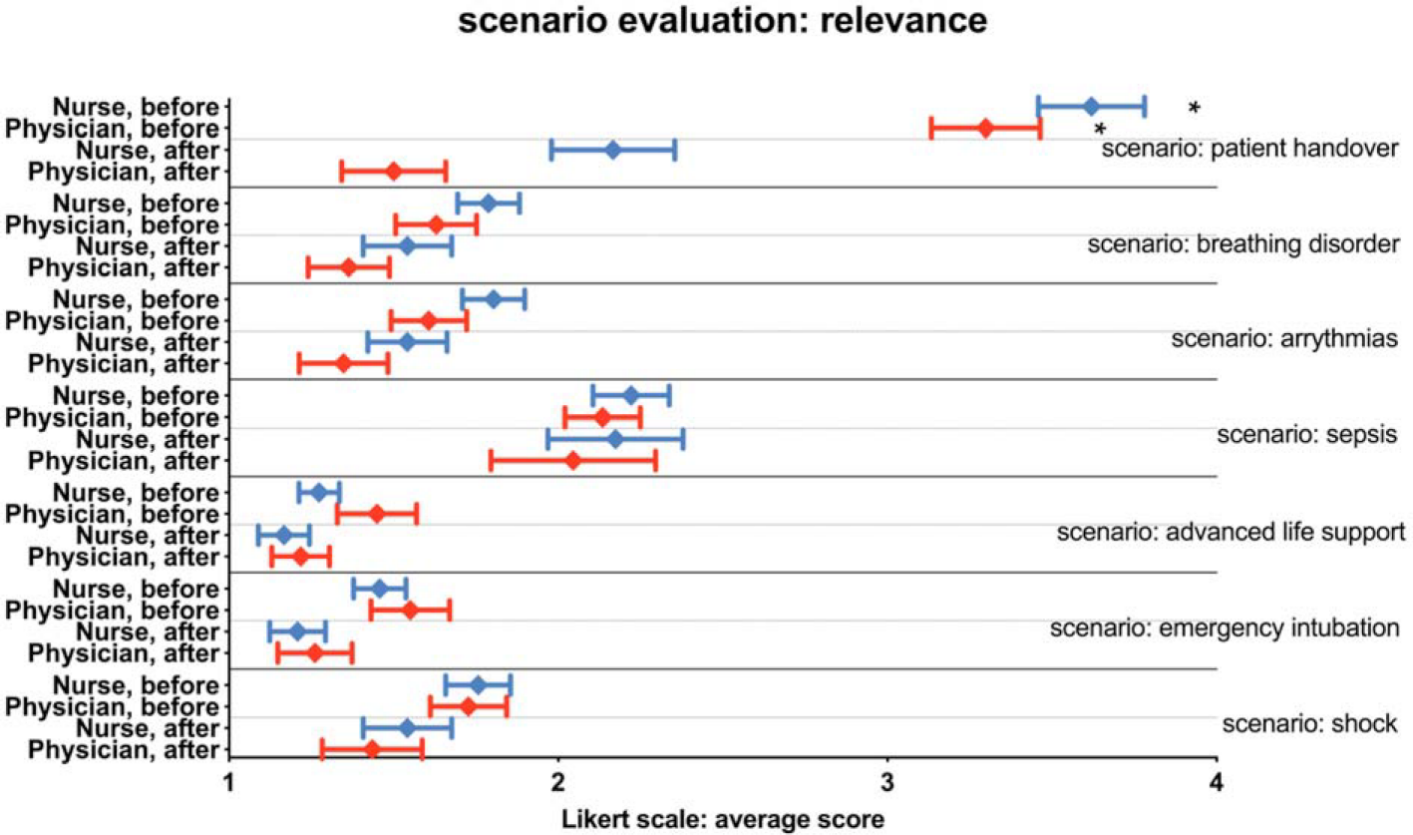
Participants’ opinions regarding the scenario before and after the course on a 6-point Likert scale (1=most useful scenario to 6= least useful scenario). The scenarios were rated very useful. The first scenario was rated significantly better (* indicates a significant difference).

The participants’ opinions regarding each scenario before and after the course appear in figure 1 (Fig. 1). On a 6-point Likert scale (1=most useful scenario to 6=least useful scenario) all scenarios were rated as very useful (values <2.11). The first scenario (patient admission) was initially viewed as being relatively unimportant, but the inclusion of hypovolaemic instability resulted in this scenario being rated quite favourably (physicians 1.50±.74, p<0.001), nurses 2.17±.92, p<0.001).

**Figure 2:**
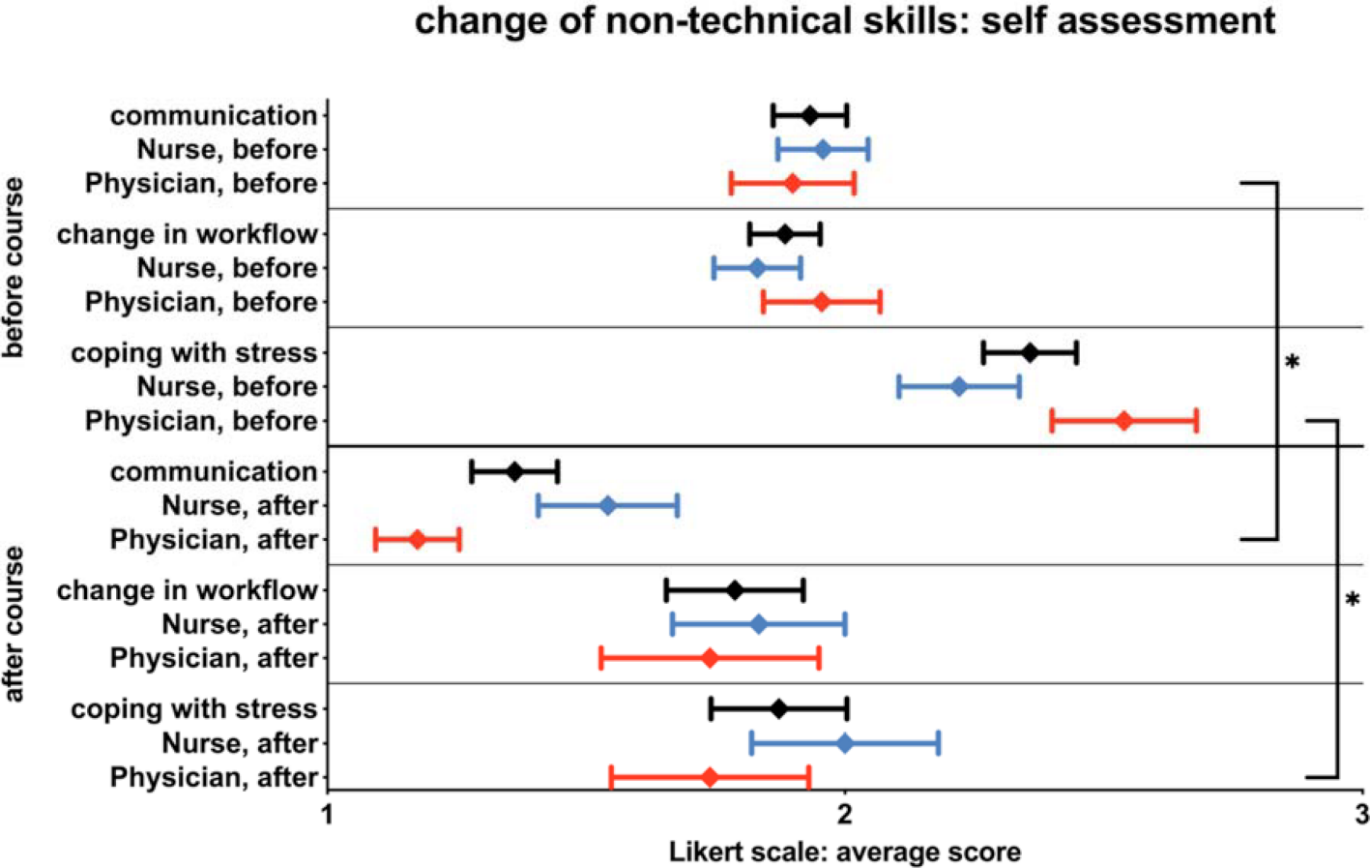
Expectation regarding the possible effects on various nontechnical skills in the own work environment on a 6-point Likert scale (1=very high relevance – 6=very low relevance). Physicians appreciate the impact on communication and coping with stress significantly better (* indicates a significant difference).

In terms of expectations regarding the assumed effects of the SBT onto various non-technical skills upon the participants’ work environment (i.e. pre-training), communication skills were expected to improve notably (physicians 1.90±1.12, nurses 1.96±.95). Workflow was expected to be improved (physicians 1.96±1.07, nurses 1.83±.91) and coping with stressful situations was expected to be influenced by the course (physicians 2.54±1.32, nurses 2.22±1.26). After the course (see figure 2 (Fig. 2)), the expected effects were rated significantly more positively by physicians for the items communication (1.17±.39, p=0.01) and stress management (1.74±.92, p=0.06). Overall, the ratings by the nurses – after completing the course – did not change significantly (communication 1.54 ± .66; workflow 1.83±.82; stress management 2.00±.89). The ratings of the item workflow by the physicians did not change significantly either (1.74±.92).

## Discussion

Our study aimed to evaluate previous exposure and experience of medical and nursing personnel with SBT. Expectations and any past experience of the participants with SBT should be incorporated into the preparation of an interprofessional crisis resource management course and its subsequent evaluation. Only 50% of the respondents had had previous exposure, which indicates that the regional coverage for this type of training is still growing. Fifteen years after “To Err is Human” and the development of an awareness of patient safety, this aspect seems interesting in that the German health care system claims to be highly developed. There might also be some misunderstanding regarding the term “simulation” as respondents also considered skill orientated ACLS (Advanced Cardiac Life Support) courses as simulation. This phenomenon has been described by Baschnegger et al. [17]. 

In the pre-training primary survey, 27% (nursing staff) and 15% (physician staff) expressed a preference not to train in mixed groups with the “opposite” profession, an attitude that needs to be gently explored and addressed by the instructors. This result reaffirms the potential for conflicts arising between the different professions in hospitals [23]. The authors themselves have, as instructors, found that interprofessional team trainings result in improved communication and cooperation, and reduce possible conflicts [24]. Advantages of interprofessional training, such as practicing exchanging mental models and practicing close-loop-communication, were discussed during the course. Thus, the question regarding mixed–profession training was not re-asked in the evaluation questionnaire. The results of the evaluation show that there was at most minor embarrassment. ”Embarrassment” was used as a quality criterion by us. The DASH-System, Debriefing Assessment for Simulation in Healthcare, specifies the psychological security of the participants as being a quality target [25]. In Germany, the concept of a protected learning environment (original: “geschütztes Lernumfeld”) was introduced for this item [26]. Overall, the training was rated as very good. Correspondingly, after completing our course, a greater majority of the participants advocated for longer training formats for this type of training. Additionally, SBTs from other ICUs (non-German-speaking countries) have similarly been rated positively by their participants [27].

As noted in our findings, the majority of nurses preferred weekdays over weekends for training courses, which likely reflects the impact that their shift working system already has upon their private time/ social life. Similarly, we have noted in our training centre, that nurses more frequently fail to attend courses for unclear reasons, predominantly on weekends. In summary, interprofessional training courses held on weekends are unlikely to be received well by teams with members whose work already infringes significantly/often on normal (socially accepted) free time.

Aspects of daily work routine are highly relevant for achieving successful learning [19]. Although all of the scenarios presented were considered appropriate and realistic by the authors, the “patient admission” scenario was initially assessed as not very suitable by the ICU staff during the pre-course assessment. Integration of haemodynamic instability into this scenario after the initial survey resulted in full acceptance. If the theoretical assessment of the scenario in the run-up to the course stands for description of learning objectives, this assessment can be classified as evaluation and feedback (Step six of the Kern cycle).

SBT in clinical anaesthesia has a positive influence on communication, confidence and teamwork [14]. Survey respondents and course participants considered the influence on communication, workflow and coping with stress as good but not excellent, thus indicating potential for improvement. These results are consistent with previous research: Haerkens et al. [28] showed that team climate, working condition and job satisfaction increase after a CRM-intervention. In addition, the work of Haerkens demonstrated a reduction in severe adverse events, e.g. cardiac arrests, as a positive result of team training seminars that incorporate crisis resource management. 

A Swedish study demonstrated that the safety attitudes questionnaire (SAQ)-Index [29], one of the most validated “safety climate” measuring tools used in healthcare, increased after simulation–based team trainings were utilized by their ICU, and furthermore, that the number of nurses quitting their jobs and nurse assistants’ time on sick leave was reduced [30]. These results are particularly interesting, as the staffing in Swedish intensive care centres is higher than German ICUs [31]. In the German healthcare system, hospitals are being run both by public and private providers; with the majority being in a tense economic situation [32]. The comparably higher costs (short term) of simulation training courses, combined with “lost” working hours, as well as the generalised shortage of professional nursing staff may hamper the implementation of simulation-based training courses. Furthermore, planning each course to consist of six doctors and six nurses for training on a regular workday will be difficult especially for smaller teams [33].

Nonetheless, our results combined with the reported potential benefits of reduced staff sick leave and turnover as well as improvements in patient care, should stimulate acceptance for the need to create these types of training courses specifically for German intensive care environments and foster attendance at them. Further, regarding the expense, Moffat-Bruce et al. showed that simulation-based training can generate a return on investment so that there is an even greater chance for change [34].

### Limitations

Limitations due to simulator technology were apparent: e.g. ultrasound was not available to differentiate hypovolaemia from cardiac malfunction in one scenario. Nonetheless, although individual technical measures may have a significant impact upon the decision-making process for a real patient’s management (i.e. if too time consuming or proving too difficult, alternative technical options will be explored in the real world) – this was not an objective for this course, rather NTS were and hence a balance was required with achieving clinical relevance for the participants without losing the focus on NTS.

Questionnaires describing learner expectancy, experience and effect on simulation training were created by a local team of experts rather than being validated in larger groups. In the run-up to the study, we had problems finding volunteer departments for the survey. We had no influence on whether and which participants of the first route participated in the course. This may have an impact on comparing the ratings. We designed the questionnaire as short as possible to get a high return rate. By the local circumstances, the subjective rating of the participants may have been an influence (e.g. culture, size of the ward).

Further demographic data such as work experience and gender were not asked because we expected the number of cases too low for statistical evaluation. There might be a lot of correlation factors between attitude to training and demographic data, work experience or type of work environment. This could be a focus of another research study. There are several questionnaires for measuring training success in CRM trainings for the German-Speaking area. Since we expected a great inexperience of the participants regarding SBT, the use of these partly complex questionnaires could deter the participants. These measurement scales from occupational psychology can be used to accompany the developed training more detailed and with an objective focus. 

Furthermore, it was not possible to evaluate the percentage of responses given by the external hospitals with respect to quantity and quality. Additionally, no differentiation was made between the main specialty overseeing the respective ICUs, e.g. anaesthesiology vs. surgery vs. internal medicine. Our training programme endeavours to enhance the relevance and acceptance of interprofessional training and, on a wider perspective, aims to improve patient safety, efficiency of treatment and caregivers’ satisfaction. To our knowledge, there are no other projects in Germany that are using SBT as an intervention for the ICU work environment in an attempt to deal with these issues.

## Conclusion

Interprofessional crisis resource management trainings using a simulator have not yet been established for ICU teams in Germany. This study investigated the expectations and needs of the core professionals, physicians and nurses, and subsequently evaluated a new SBT course format designed specifically for ICUs. The course itself was well received and evaluated favourably. Our work is the first step in gaining the acceptance for and subsequently the integration of these teaching strategies into the high responsibility environment of German ICUs. The next steps will involve evaluating the actual impact they have on the work environment, including patient safety, sick leave and team cohesion.

## Competing interests

The authors declare that they have no competing interests. 

## Figures and Tables

**Table 1:**
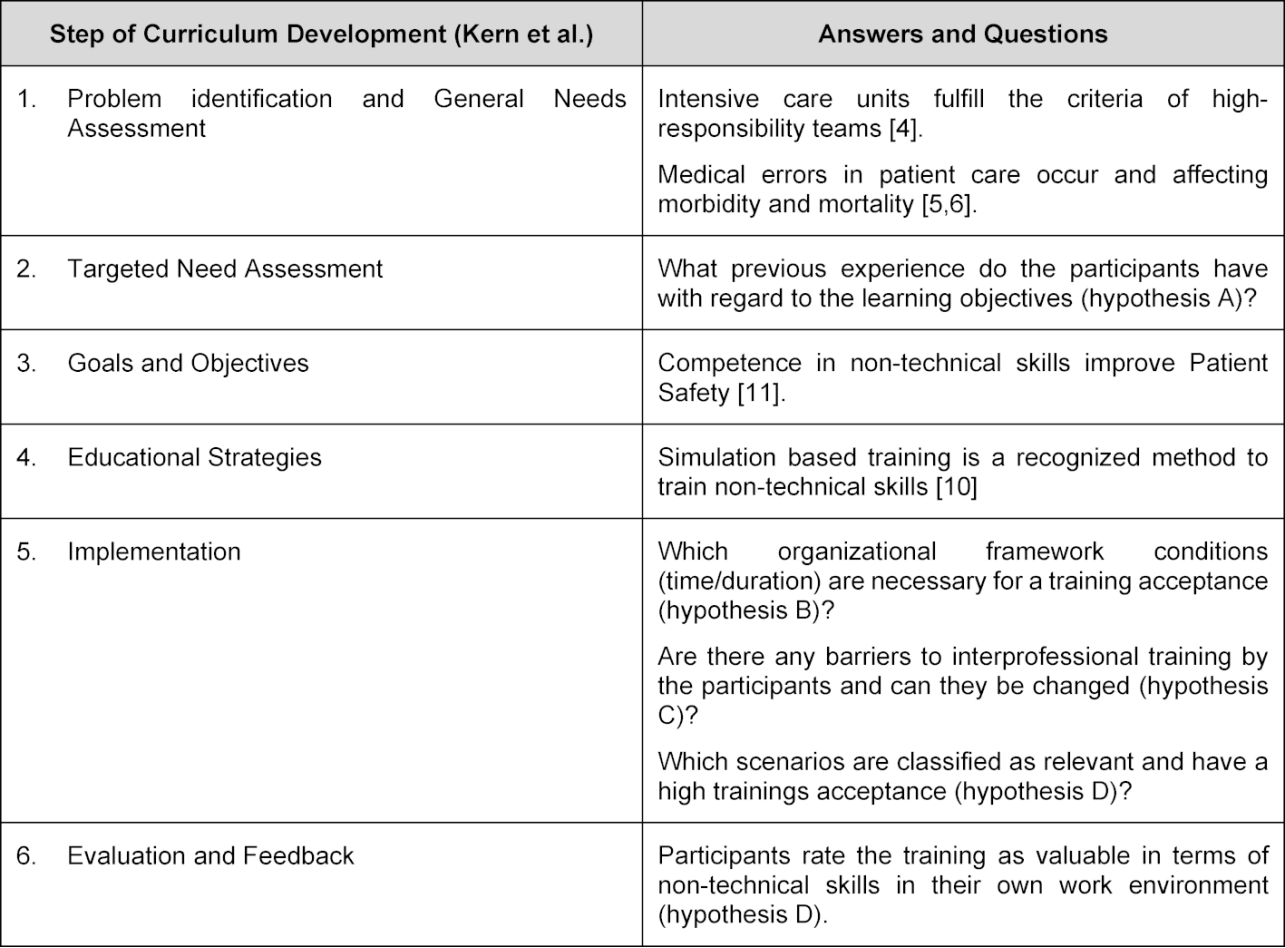
Six-Step Approach by Kern et. al. and application to the development of a relevant training concept for intensive care units.

**Table 2:**
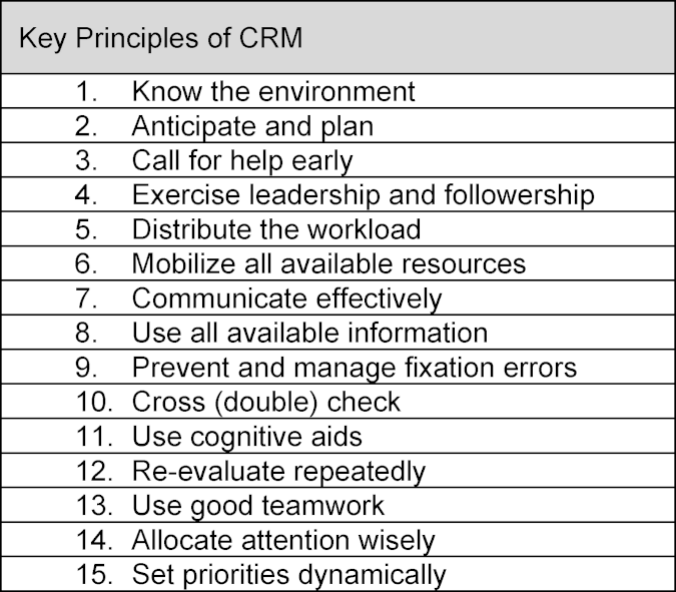
The 15 key principles of CRM (crisis resource management) as per Gaba and Rall [22].

**Table 3:**
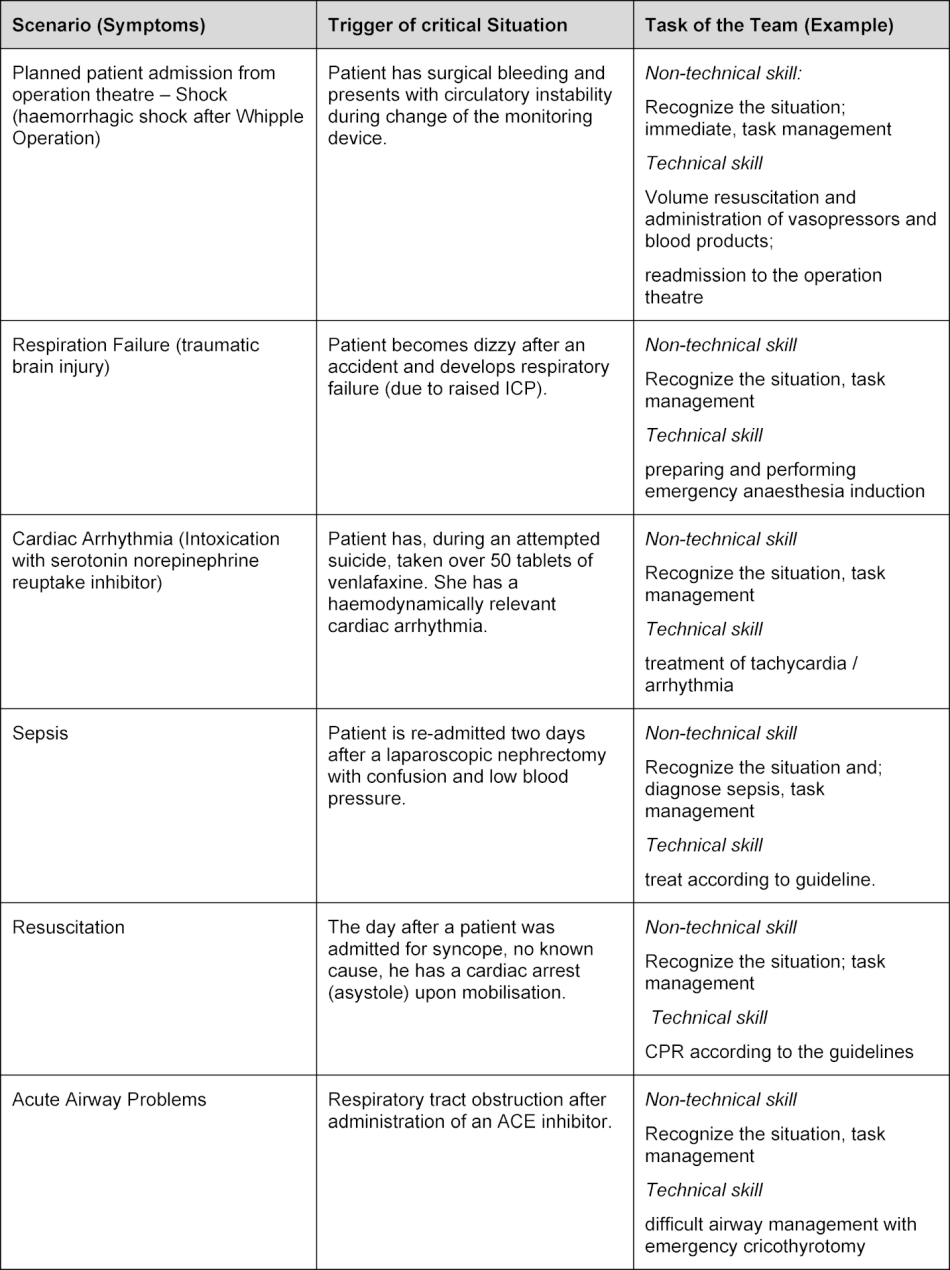
Scenarios and stress trigger in the simulation course. The scenarios Patient Admission and Shock were combined after the first survey. Each scenario was tackled interprofessional, by a nurse with a physician.

**Table 4:**
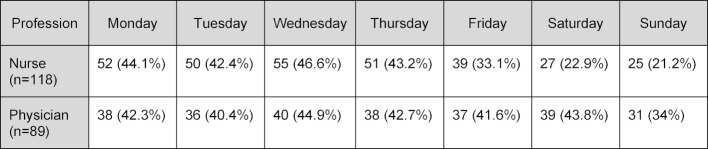
Preferred day for the courses (more than one day could be given as desirable). Physicians were more willing to train on weekends. Percentages of absolute numbers are given in parenthesis.
